# External Validation of a Risk Model for Severe Complications following Pancreatoduodenectomy Based on Three Preoperative Variables

**DOI:** 10.3390/cancers14225551

**Published:** 2022-11-11

**Authors:** Zahraa M. Alhulaili, Rick G. Pleijhuis, Maarten W. Nijkamp, Joost M. Klaase

**Affiliations:** 1 Department of Hepato-Pancreato-Biliary Surgery and Liver Transplantation, University Medical Center Groningen, University of Groningen, P.O. Box 30001, 9700 RB Groningen, The Netherlands; 2 Department of Internal Medicine, University Medical Center Groningen, University of Groningen, P.O. Box 30001, 9700 RB Groningen, The Netherlands

**Keywords:** pancreatoduodenectomy, complications, CT scan, Whipple, PPPD, risk prediction model

## Abstract

**Simple Summary:**

Up to 30% of patients develop severe complications following pancreatoduodenectomy (PD). With respect to risk stratification and shared decision making, prediction models to predict complications are crucial. In 2015, a risk model for severe complications was developed by Schroder et al. based on three preoperative variables: BMI, ASA classification and mean Hounsfield Units of the pancreatic body on the preoperative abdominal CT scan. However, external validation of this model has not yet been performed. It is important to validate prediction models externally before implementing them in clinical practice to confirm their accuracy and generalizability when applied to a different patient population. Our aim was to externally validate this risk prediction model using an independent cohort of patients.

**Abstract:**

Background: Pancreatoduodenectomy (PD) is the only cure for periampullary and pancreatic cancer. It has morbidity rates of 40–60%, with severe complications in 30%. Prediction models to predict complications are crucial. A risk model for severe complications was developed by Schroder et al. based on BMI, ASA classification and Hounsfield Units of the pancreatic body on the preoperative CT scan. These variables were independent predictors for severe complications upon internal validation. Our aim was to externally validate this model using an independent cohort of patients. Methods: A retrospective analysis was performed on 318 patients who underwent PD at our institution from 2013 to 2021. The outcome of interest was severe complications Clavien–Dindo ≥ IIIa. Model calibration, discrimination and performance were assessed. Results: A total of 308 patients were included. Patients with incomplete data were excluded. A total of 89 (28.9%) patients had severe complications. The externally validated model achieved: C-index = 0.67 (95% CI: 0.60–0.73), regression coefficient = 0.37, intercept = 0.13, Brier score = 0.25. Conclusions: The performance ability, discriminative power, and calibration of this model were acceptable. Our risk calculator can help surgeons identify high-risk patients for post-operative complications to improve shared decision-making and tailor perioperative management.

## 1. Introduction

Pancreatoduodenectomy (PD) is the operative management for patients with pancreatic head cancer and malignant or premalignant periampullary pathologies [1,2]. The mortality rates following this surgery have decreased over time to less than 5% [2,3,4,5]. This reduction in mortality rates is due to the improvements in perioperative management and the advances in surgical skills and procedures in combination with centralization [2,6]. The indications for this operation have been expanded to incorporate benign pathologies which may reflect on the increase of survival rates as well [5]. Despite the reduction in mortality rates, the overall complication rates remain relatively high, ranging from 40–60%, with up to 30% with severe complications [1,4,5,7,8].

Many severe complications can occur following PD, the most frequently mentioned in the literature are post-operative pancreatic fistula (POPF), delayed gastric emptying (DGE) and post-pancreatectomy hemorrhage (PPH). All three complications are classified according to the International Study Group of Pancreatic Surgery (ISGPS) classification system [9]. DGE is the most common complication following PD with an incidence rate of 20–50% [3,10]. POPF is a serious and life-threatening complication with incidence rates between 5 and 40% [3]. POPF is graded according to the ISGPS definition in which grade A, also known as biochemical leakage, is not clinically relevant [11]. Grades B and C result in negative consequences for the patient [11]. PPH and deep surgical site infection are complications that can be related to POPF with incident rates from 3% to 16% and from 12 to 51%, respectively [12,13]. Less frequently occurring is bile duct leakage [5]. 

To classify the severity of complications after gastrointestinal surgical procedures, the Clavien–Dindo classification system is commonly used [14]. This classification consists of five grades in which grades three and four are further subdivided into A and B subgrades, and grade five indicates mortality [3,14]. Each grade depends on the required intervention to resolve the complications, the higher the grade is, the more complex the intervention needed [3,14]. In the present study, severe complications were defined as grade IIIa or higher. This means that the complications at least demand surgical, radiological, or endoscopic interventions under local anesthesia [3,14]. There are multiple pre-operative risk factors that may increase the occurrence of complications, such as advanced age, high body mass index (BMI), male gender, high American Society of Anesthesiology (ASA) classification, and comorbidities such as diabetes and cardiovascular disease [15,16,17]. The most important intra-operative risk factors are a soft pancreatic texture, a small pancreatic duct less than 3 mm, intra-operative blood loss, pancreatic steatosis and/or the absence of pancreatic fibrosis [17,18,19]. 

Several risk calculators for pancreatic surgery have been developed over the past decades. Most researchers focused with their risk calculators on survival or POPF [20,21,22]. Nevertheless, there are in general very limited risk calculators for severe complications [1]. For the individual patient the risk of severe complications (Clavien–Dindo classification IIIa or more) outweighs the risk of POPF only. The risk calculators, especially when they are based on pre-operatively known risk factors, can be used during the informed consent process [2]. It is also important for the surgeon to make a well-considered decision regarding an individual’s surgical risks and evaluate whether the benefits for such an operation outweighs the risks [2]. Several precautions can be taken, such as, additional preoperative assessment, choosing the most suitable reconstruction technique, careful post-operative monitoring, and better preparation for other possible complications [6,8,23]. Another important argument to estimate the post-operative risk is to reduce the adverse consequences of the complications. It is known that complications can lead to multiple organ failure or mortality, prolong hospital stay and/or required readmission and increased costs [24,25,26]. A complicated course after pancreatoduodenectomy may lead to impaired long-term survival [27].

Recently, a risk model for severe complications after PD was developed by Schroder et al. based on three preoperative variables: ASA classification, BMI, and mean Hounsfield Units (HU) of pancreatic body on the preoperative CT scan [28]. The authors reported that high BMI, ASA III classification and low mean Hounsfield Units of the pancreatic body on the preoperative abdominal CT scan were independent predictors for severe complications upon internal validation. However, no external model validation has yet been performed to confirm the robustness and generalizability of this model. In the current study, we sought to externally validate the above-mentioned risk model using an independent and larger cohort of patients treated at our institution.

## 2. Materials and Methods

### 2.1. Study Design and Study Population

The present study is a retrospective cohort study. Patients who underwent pancreatic surgery were registered in the mandatory national registration of pancreatic surgery, the Dutch Pancreatic Cancer Audit (DPCA) database. This electronic database was used in our study. The medical records of 318 consecutive patients who underwent pancreatoduodenectomy (PD) or pylorus-preserving pancreaticoduodenectomy (PPPD) at the University Medical Center Groningen in the Netherlands between the periods January 2013 and December 2021 were reviewed. 

### 2.2. Data Collection and Endpoint Definition

The following data were collected for each patient: age, gender, Body Mass Index (BMI), American Society of Anesthesiologist (ASA) classification, date of operation, type of operation, date of the pre-operative abdominal CT scan, CT scan phase, slice thickness, and Hounsfield Units (HU) of the pancreatic body. The BMI and ASA classification were identified from the pre-operative anesthesiology report. HU of the pancreatic body were measured on pre-operative contrast enhanced portal venous CT scans using a region of interest (ROI) greater than 1 cm without involvement of the pancreatic duct or any of the adjacent blood vessels. Slice thicknesses ranged between 0.75 and 5.00 mm. 

**Figure 1:**
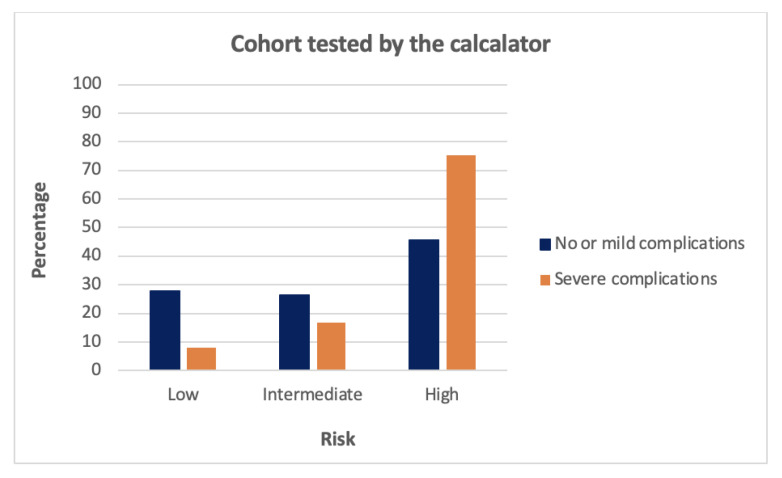
Results obtained from the online test of our cohort (*n* = 308) with the calculator. It represents the percentage of patients within each category and whether the risk acquired by the calculator was low, intermediate, or high.

Complications were scored using the Clavien–Dindo classification system. In our study, the outcome of interest was the occurrence of severe complications defined as Clavien–Dindo ≥ IIIa within 30 days after surgery. Both the researcher and the data analyzer were blinded for the outcome of interest. The Clavien–Dindo classifications were made available to the researcher when all measurements were completed. The sample was then tested by the online calculator and three risk groups were obtained, namely low, intermediate, and high (Figure 1). The same applied to data validation. An anonymized dataset was used by the data analyzer to validate the study. 

### 2.3. Statistical Analysis

Statistical analysis of the study population was conducted using SPSS Statistics (IBM Corp., version 28, Armonk, NY, USA). Continuous variables were reported as mean ± standard deviation (SD) when the variables were normally distributed or as median and interquartile ranges (IQR) when the variables were non-normally distributed. Categorical variables were expressed as percentages and frequencies. A *p* value was calculated using a two-tailed *t*-test and a *p* < 0.05 was considered statically significant. To validate the prediction model, the collected anonymized data of the validation cohort was entered into the Evidencio platform (Evidencio BV, version 3.12, Haaksbergen, The Netherlands). This platform facilitates external validation through an online semi-automated validation tool, calculating multiple model performance parameters simultaneously [29]. 

### 2.4. Evaluated Parameters of Model Performance

Calibration was assessed both visually (calibration plot) and through calculation of the slope and intercept, evaluating the level of agreement between predicted probabilities versus observed outcomes. Model discrimination was assessed by calculating the area under the receiver-operating characteristic curve (C-statistic with 95% confidence interval). A classification plot was generated to plot sensitivity versus 1-specificity. Overall model performance was evaluated using the Brier score as a composite measure of both model discrimination and calibration. The scaled Brier score was calculated to take the baseline prevalence of severe post-operative complications into account.

### 2.5. Ethics

This study falls within the scope of the non–WMO system. The Dutch Medical Research with Human Subjects Law is not applicable to this study. All other necessary steps have already been completed. There was no need for approval from the Medical Ethical Evaluation Committee (METc) at our hospital.

## 3. Results

### 3.1. Baseline Characteristics of the Patients 

A total of 308 patients who underwent PD or PPPD at our institution and for whom data was complete were included. Ten patients were excluded due to the presence of inappropriate or incomplete CT scans. There were no other exclusion criteria applied, such as vascular resection, borderline tumor and neoadjuvant therapy. The median age of the study cohort was 67 years (range 59–73), consisting of slightly more males (52.9%, *n* = 163) than females. PPPD was performed in 245 patients (79.5%), whereas PD in 63 patients (20.5%). The majority of the patients had ASA II classification (64.3%, *n* = 198) and the mean BMI was 26.6 ± 11.8. The median range of HU of the pancreatic body as measured on a pre-operative CT scan was 85.3 (range 70.7–96.4) with lower ranges in those patients who had severe complications being 79.6 (range 62.9–89.9). The most common pathology was adenocarcinoma (57.1%, *n* = 176), followed by intestinal type adenoma and neuroendocrine neoplasm (11.7%, *n* = 36 and 7.8%, *n* = 24, respectively). Severe complications defined as Clavien–Dindo IIIa or higher occurred in 89 (28.9%) patients. Baseline characteristics of this validation cohort are reported in Table 1. 

**Figure 2:**
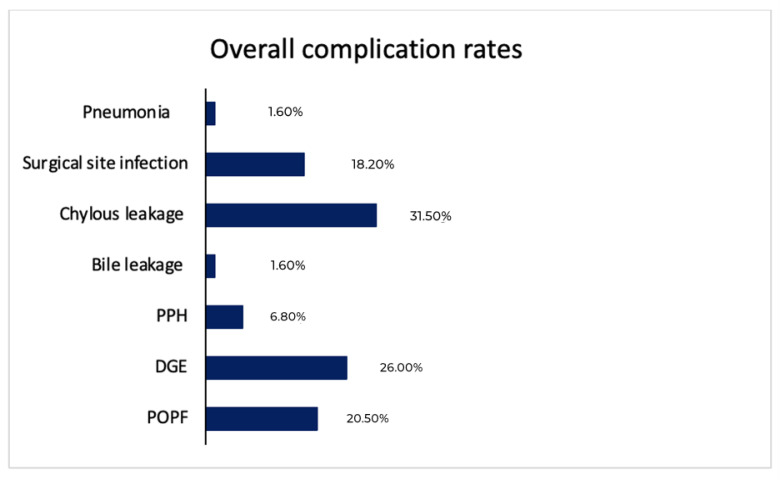
Overall morbidity and their rates in the validation cohort (*n* = 308) patients.

The most common complication in our cohort was chylous leakage followed by DGE, 31.5% and 26%, respectively. Chylous leakage was mainly grade A (19.5%, *n* = 60), whereas DGE was mainly grades A or B (10.4%, *n* = 32 and 10.7%, *n* = 33, respectively). The superficial surgical site infection rate was 18.2% (*n* = 56). There were 44 patients who developed clinically relevant POPF, grade B (12.7%, *n* = 39) or grade C (1.6%, *n* = 5). The incidence of PPH grade B was more than grades A and C (3.6%, *n* = 11, 2.6%, *n* = 8 and 0.6%, *n* = 2, respectively). Pneumonia and bile leakage were rare in our cohort, only 10 patients had either of these complications. Mortality rate was 1.6 (*n* = 5). Observed post-operative complications are summarized in Table 2 and Figure 2. 

There were similarities and differences in some variables observed in the validation cohort in comparison to the development cohort. Both cohorts were comparable in terms of age (65 ± 10.6 and 66 ± 9.3, respectively), more males (52.9%, *n* = 163 and 61.8%, *n* = 68, respectively), HU of the pancreatic body (85.3 (70.7–96.4) and 79.4 (61.1–92.9), respectively), more ASA II (64.3%, *n* = 198 and 90.9%, *n* = 100), and BMI (26.6 ± 11.8 and 25 ± 3.7, respectively). However, PPPD was performed less in the development cohort, namely 64.5% compared to 79.5% in the validation cohort. Severe complications occurred more frequently in the development cohort compared to the validation cohort, 33% and 28.9%, respectively.

### 3.2. Model Discrimination

**Figure 3:**
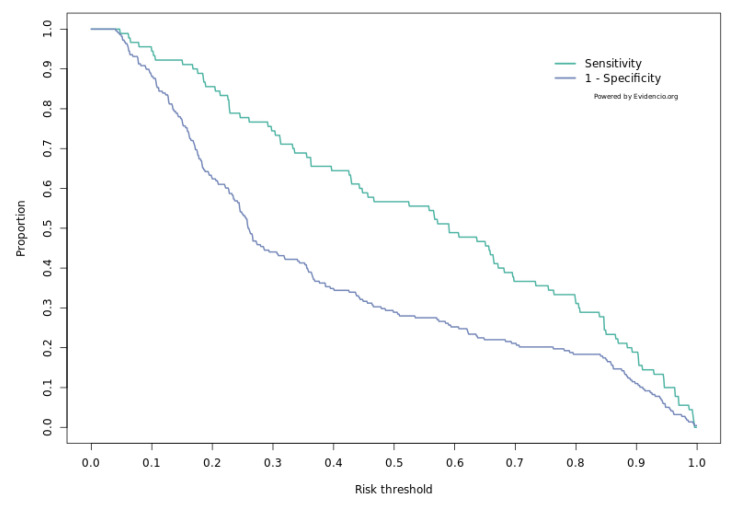
Classification plot representing the relationship between sensitivity and 1-specificity.

Our model demonstrated a C-statistic of 0.67 (95% confidence interval (CI): 0.60–0.73). The C-index can range between 0.5 and 1.0, in which values of 0.5 to 0.6, 0.6 to 0.7, 0.7 to 0.8, and 0.8 to 0.9 indicate poor, fair, moderate, and good model discrimination, respectively, while a value of 1.0 implies perfect discrimination. In our study C-statistic of 0.67 implies fair discrimination. A classification plot was generated to display the sensitivity and 1-specificity of the model (Figure 3). 

### 3.3. Model Calibration 

**Figure 4:**
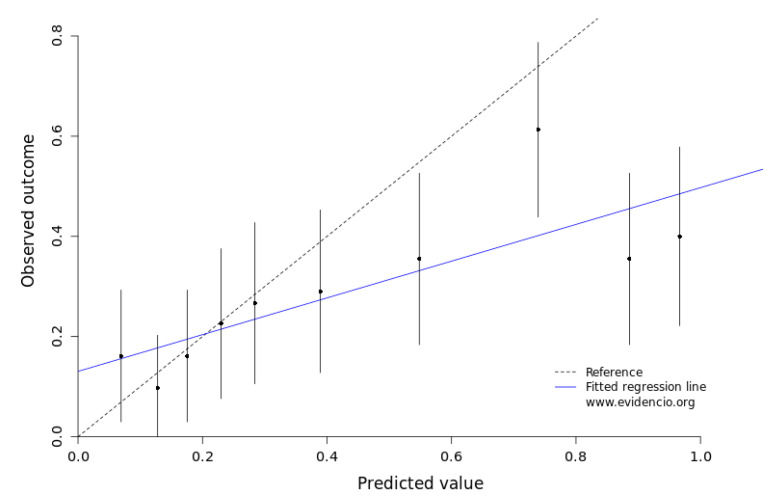
Graphical representation of model calibration, plotting the predicted probability (*x*-axis) with corresponding 95% CI against the actually observed occurrence of severe post-operative complications (defined as Clavien–Dindo IIIa or higher) in the validation cohort. The dashed line represents perfect calibration. Points under the line display overestimation, and above the line underestimation of the risk of severe post-operative complications.

A calibration plot was generated in which the *x*-axis represents the predicted and the *y*-axis the observed severe complication rates (Figure 4). This model had a slope of 0.37 and an intercept of 0.13 representing an acceptable model.

### 3.4. Overall Model Performance

The validated model achieved a Brier score of 0.25, with a score of 0 suggesting good accuracy and a score of 1 suggesting inaccuracy of the model. The calculated scaled Brier score was <0.001. This indicates moderate accuracy of the model.

## 4. Discussion

The aim of the present study was to externally validate the risk model for severe complications after PD developed by Schroder et al. consisting of three variables: BMI, ASA classification and mean HU of the pancreatic body on the pre-operative abdominal CT scan. Our validation cohort consisted of 308 patients who underwent PD or PPPD at our institution. Baseline characteristics were comparable between the development and validation cohorts. Severe complications occurred more often in the development cohort, namely 33%. In our study the severe complication rate was 28.9%, which is lower than other studies [1,23]. Assessment of the model’s performance parameters resulted in a C-index of 0.67, a regression slope of 0.37, an intercept of 0.13, a Brier score of 0.25, and a scaled Brier score of <0.001. Based on these parameters, we considered it a fair risk model for prediction of severe complications.

The risk of developing pancreatic cancer is higher amongst elderly people [30]. As the population is aging, more people are developing pancreatic or periampullary cancer which can only be cured with surgery. A complex procedure such as PD with a severe complication risk rate up to 30% is more dangerous at advanced age due to the presence of comorbidities, such as cardiovascular diseases and diabetes mellitus [31]. A pre-operative risk calculator for severe complications may help to identify high-risk patients, thereby guiding clinical decision-making. Prehabilitation might also be effective in patients with comorbidities [32]. Frail patients can undergo a prehabilitation program to increase their general fitness and anerobic threshold while awaiting the surgery. This could decrease the prevalence of post-operative complications [33]. Moreover, the surgical technique can be adjusted, for example, an isolated roux loop for the pancreatic anastomosis can be used separating the pancreatojejunostomy and hepatojejunostomy [33]. 

The majority of the published risk models use one or more intra-operative risk factors, for example, pancreatic texture and intra-operative blood loss. Our risk calculator is unique in this respect as it only requires pre-operative variables. ASA classification and BMI can be obtained from the anesthesiology report which is made during the pre-operative assessment. The HU of the pancreatic body can be easily measured on the pre-operative CT scan. All measurements can be performed by the surgeons themselves and it does not require assistance from a radiologist nor special applications. All measurements can be conducted on the desktop. When all variables are available, they can be entered into the online calculator. Based on their predicted individual risk, patients are than stratified into one of three categorical risk groups: low, intermediate, or high.

Compared with other risk prediction models, this model was developed to estimate the risk of severe complications following PD. Most existing models evaluate only the risk of POPF and do not involve other complications. Although POPF is a serious complication, focusing on the POPF risk alone would lead to a significant underestimation of complication rates. In addition, an adequate explanation of the risk model and how it can be used by surgeons is not always specified. Furthermore, there were few risk models that used the pre-operative CT scan to predict post-operative complications without developing a risk calculator [34,35]. Notably, some researchers studied the role of the pre-operative CT scan to predict the risk of POPF alone [36,37,38,39,40]. Table 3 provides a summary of the risk calculators that were established to estimate the risk of severe complications after PD. 

Amongst the risk calculators that focus on all types of severe complications is the Braga calculator [4]. It has been externally validated in the Joliat G et al. series and it showed a good accuracy with a C-index of 0.99, Hosmer–Lemeshow test *p* = 0.82 [23]. Despite good accuracy, this study has some limitations, as it involved only patients who had PD as a resection technique and not PPPD. The results may have been biased because the Clavien–Dindo classification was published in 2004 and these two studies included patients before this period. Moreover, this calculator uses many intra-operative variables which makes it unsuitable for use during the pre-operative phase. The pancreatoduodenectomy risk tool published by Abbas A et al. is available online [24]. It showed a C-index for severe complications of 0.63; however, no model calibration measure was reported, and this study also has several limitations. The model requires many variables in which pancreatic texture was considered as a pre-operative variable and evaluated based on the type of pathology. Likewise, no clear classification was provided in terms of severe complications and their definitions. The risk model for complications established by Chen L et al. also involves pancreatic texture as an intra-operative variable, and it is graded subjectively during the operation [1]. Clavien–Dindo of II or higher was considered significant, whereas most studies reported in the literature use alternative cut-offs (IIIa, IIIb, or higher) [2,4,8,23,28]. The PREPARE calculator was developed by Uzunoglu F et al. and validated in Rodriguez-Lopez M et al. series [41,42]. It demonstrated a moderate accuracy with an AUC of 0.71 (95% CI: 0.56–0.87) and Hosmer–Lemeshow, *p* = 0.86, yet the validation cohort incorporated a small number of patients (*n* = 50), there were patients who underwent distal pancreatectomy, and it was not mentioned how many patients had this type of resection. This might have had an impact on the outcomes because this type of resection is associated with a lower morbidity rate [41,42]. The risk calculator developed by Wiltberger G et al. used preoperative variables and there was no pancreas specific variable [16]. It has not been validated yet as well [16]. The risk model of Aoki S et al. involved 20 pre-operative variables and one intra-operative variable (vascular resection) [2]. Severe complications were defined as Clavien–Dindo ≥ IV and the AUC was 0.71 (95% CI: 0.69–0.73). Again, no calibration measure was reported, and no external validation has been performed yet.

There were many studies established using the American College of Surgeons-National Surgical Quality Improvement Program (ACS-NSQIP) database. For example, a procedure-specific ACS-NSQIP was developed by McMillan M et al. and comparable to the non-procedure-specific ACS-NSQIP calculator for general surgery [43]. It differs in using fistula Risk score (FRS), hospital volume and surgical skills as additional predictors for complication risk. The C-statistic was 0.65, Brier score 0.19, and Spiegelhalter’s z-test *p* = 0.189. Nevertheless, FRS calculation requires intra-operative blood loss and pancreatic texture which makes it inappropriate for pre-operative usage. Another pancreas specific calculator is the ACS-NSQIP pancreatoduodenectomy risk calculator which uses 10 preoperative variables [7]. The authors reported a C-statistic of 0.61 upon internal validation of the model, while no external validation was performed. In comparison, we found a C-statistic of 0.67 for our model when assessed in an independent patient cohort. Furthermore, the ACS-NSQIP uses many variables and involved approximately 34% distal pancreatectomies which may reduce the reliability of the outcomes. The study by Greenblatt D et al. used the same database [44]. It requires many variables in which no pancreas specific variable was used, and severe complications were not defined according to the Clavien–Dindo definition.

### Strengths and Limitations

We have successfully validated the novel model to predict severe complications after PD. Model discrimination and calibration were considered fair. An important strength of our study was the simultaneous evaluation of multiple model performance measurements, providing a comprehensive indication of overall model performance [45]. Another strength is that our model is unique and easy to use. The necessary instructions for the calculation are available in the online calculator. Moreover, in this study the researchers were blinded while collecting and analyzing the data to prevent bias and to acquire trustworthy validation outcomes.

To our knowledge, this is the first risk calculator that incorporates pre-operative CT scans in estimating the risk of severe complications. Until present, researchers have used the pre-operative CT scan in developing calculators for POPF alone and not all severe complications together [37,40]. HU of the pancreatic body on a portal venous CT scan is a measure of the fatty content of the pancreas. This means that if the HU is low, there will be more fat present. It is known that a fatty pancreas increases the risk of complications [28]. However, until today this can only be evaluated on CT or MRI scans pre-operatively or subjectively during the operation. It is remarkable that, until now, imaging scans, which are always available before surgery, are not frequently used to measure the pancreatic texture. Furthermore, our calculator includes BMI, which is a morbidity measure, ASA classification which is an indicator for the patient’s condition to tolerate surgery, and a pancreas specific variable (HU of the pancreatic body).

The current study has several limitations. First, we performed a retrospective validation based on a single center cohort of patients. This may result in a selection bias because each hospital has specific protocols when deciding which patients can undergo the surgery and while selecting the reconstruction technique. Second, this score is developed and validated to predict all possible complications based on severity lacking the ability to predict a specific complication. Therefore, it is difficult to estimate beforehand the type of complications that may occur. Third, the development study did not quantify the type of complications that occurred post-operatively, making it difficult to compare the two cohorts in this respect.

Our risk calculator is validated on patients who underwent an open PD or PPPD. We recommend further validation of our model on patients who underwent robotic-assisted PD, as we believe that this procedure will be performed more in the future. Finally, we would like to encourage future researchers to validate the existing prediction models instead of developing new ones. External validation is considered an essential step prior to actual application of prediction models in the clinical practice. This applies to our model as well. We invite other researchers to validate our model in different group of patients.

## 5. Conclusions

Our risk model was superior to most of the established risk models predicting severe complications after PD. Assessment of the different model parameters of discrimination, calibration and performance indicated an acceptable overall model for predicting severe complications. Our model can be used by surgeons to predict the risk of severe complications and take the necessary measures in perioperative care of their patients.

## Figures and Tables

**Table 1:** Baseline characteristics of the study cohort.

	All (308)	No or Mild Complications Clavien–Dindo (I, II) (219)	Severe Complications Clavien–Dindo ≥ IIIa (89)	*p* Value
Age (years)				0.358
Median (IQR)	67 (59–73)	67 (58–73)	67 (63–72)	
Mean ± SD	65 ± 10.6	65 ± 11.5	66 ± 8.2	
Gender, *n* (%)				0.348
Male	163 (52.9%)	118 (53.9%)	45 (50.6%)	
Female	145 (47.1%)	101 (46.1%)	44 (49.4%)	
BMI (kg/m^2^) Median (IQR)	25.4 (22.4–28.7)	25.1 (22.3–27.9)	26.4 (23–30)	0.638
Mean ± SD	26.6 ± 11.8	26.5 ± 13.7	27 ± 5.1	
(25 *n* (%))	138 (44.8%)	108(49.3%)	30 (33.7%)	
(25 *n* (%))	170 (55.2%)	111(50.7%)	59 (66.3%)	
ASA score, *n* (%)				0.330
I	31 (10.1%)	21 (9.6%)	10 (11.2%)	
II	198 (64.3%)	152 (69.4%)	46 (51.7%)	
III	79 (25.6%)	46 (21%)	33 (37.1%)	
HU of the pancreas				0.635
body, Median (IQR)	85.3 (70.7–96.4)	87.6 (72.7–98.7)	79.6 (62.9–89.9)	
Mean ± SD	84.6 ± 17.9	87.2 ± 17.6	78.2 ± 17.3	
Pathology				0.556
Adenocarcinoma	176 (57.1%)	134 (61.2%)	42 (47.2%)	
IPMN	16 (5.2%)	12 (5.5%)	4 (4.5%)	
Neuroendocrine neoplasm	24 (7.8%)	18 (8.2%)	6 (6.7%)	
Adenoma intestinal type	36 (11.7%)	20 (9.1%)	16 (18%)	
Mucinous cystic neoplasm	6 (1.9%)	4 (1.8%)	2 (2.2%)	
Serous cystadenoma	2 (0.6%)	1 (0.5%)	1 (1.1%)	
Chronic pancreatitis	5 (1.6%)	5 (2.3%)	0	
Chronic cholangitis	9 (2.9%)	7 (3.2%)	2 (2.2%)	
Other/Unknown	34 (11%)	18 (8.3%)	16 (17.9%)	
Operation type *n* (%)				0.825
PD	63 (20.5%)	48 (21.9%)	15 (16.9%)	
PPPD	245 (79.5%)	171 (78.1%)	74 (83.1%)	

Hounsfield Units (HU), Body Mass Index (BMI), American Society of Anesthesiologist (ASA), Pancreatoduodenectomy (PD), Pylorus-preserving pancreatoduodenectomy (PPPD), Standard deviation (SD), Interquartile range (IQR), Intraductal Papillary Mucinous Neoplasm (IPMN).

**Table 2:** Postoperative complications in detail.

Complication	*n*	%
POPF		
No	245	79.5
Grade A	19	6.2
Grade B	39	12.7
Grade C	5	1.6
DGE		
No	228	74
Grade A	32	10.4
Grade B	33	10.7
Grade C	15	4.9
PPH		
No	287	93.2
Grade A	8	2.6
Grade B	11	3.6
Grade C	2	0.6
Bile leakage		
No	303	98.4
Grade A	0	0
Grade B	5	1.6
Grade C	0	0
Chylous leakage		
No	211	68.5
Grade A	60	19.5
Grade B	37	12
Grade C	0	0
Surgical site infection		
No	252	81.8
Yes	56	18.2
Pneumonia		
No	303	98.4
Yes	5	1.6
Mortality		
No	303	98.4
Yes	5	1.6

Post-Operative Pancreatic Fistula (POPF), Delayed Gastric Emptying (DGE), Post-Pancreatectomy Hemorrhage (PPH).

**Table 3:** A summary of the established risk calculators focusing on postoperative severe complications following pancreatoduodenectomy.

Study ID	Variables	Types of Variables	Severe Complication Definition	Limitations of the Calculator
#Braga et al. [4]	ASA classification* pancreatic texture, interoperative blood loss, pancreatic duct diameter.	Pre-operative intraoperative	Clavien–Dindo ≥ IIIa	Many intraoperative variables. The results may have been biased because the Clavien–Dindo was published in 2004 and it included patients between 2002 and 2010.
√Joliat et al. [23]	ASA classification* pancreatic texture, interoperative blood loss, pancreatic duct diameter.	Pre-operative intraoperative	Clavien–Dindo ≥ IIIa	Many intraoperative variables. It only included PD and not PPPD.The results may have been biased because the Clavien–Dindo was published in 2004 and it included patients between 2000 and 2012.
#Abbas et al. [24]	age, gender, BMI*, ASA classification* hypertension, gland texture, duct size, DM* adenocarcinoma.	Pre-operative	No clear classification has been provided	Difficult to use. Requires many variables. Pancreatic texture was considered as a preoperative variable. No model calibration measure was reported.
#Chen et al. [1]	BMI*, pancreatic texture, WBCs*, sodium concentration respiratory diseases	Pre-operative Intra-operative	Clavien–Dindo ≥ II	Pancreatic texture is an intraoperative variable, and it is graded subjectively during the operation.
#Uzunoglu et al. [41]	ASA classification* albumin, hemoglobin, heart rate, blood pressure, origin of tumor, elective surgery, type of operation.	Pre-operative	Clavien–Dindo ≥ III	Validated in a small cohort (*n* = 50).
√Rodriguez-Lopez et al. [42]	ASA classification* albumin, hemoglobin, heart rate, blood pressure, origin of tumor, elective surgery, type of operation.	Pre-operative	Clavien–Dindo ≥ III	Small number of patients (*n* = 50). There were patients who underwent distal pancreatectomy, and it was not mention how many patients had this type of resection.
#Wiltberger et al. [16]	BMI*, ASA classification*the presence of vascular or pulmonary comorbidities.	Pre-operative	Clavien–Dindo ≥ IIIb	No external validation. No pancreas specific variable.
#Aoki et al. [2]	20 preoperative variables, one intraoperative variable (vascular resection)	Pre-operative Intra-operative	Clavien–Dindo ≥ IV	No external validation. Requires an intraoperative variable. Not available online. No model calibration measure was reported.
#McMillan et al. [43]	The same variables as the ACS-NSQIP* calculator. It used FRS*, hospital volume and surgical skills as additional predictors.	Pre-operative Intra-operative	Accordion grade ≥ III	FRS calculation requires intraoperative blood loss and pancreatic texture. Requires many variables. Severe complications were not defined according to the Clavien–Dindo definition.
#Parikh et al. [7]	Age, gender, BMI*, history of sepsis, functional status, ASA classification* CHD*, dyspnea, bleeding disorder, type of resection	Pre-operative	Defined depending on the complication. Examples are provided in the article.	Many patients underwent distal pancreatectomy 34%. Requires many variables. Severe complications were not defined according to the Clavien–Dindo definition.
#Greenblatt et al. [44]	Age, gender, BMI, functional status, COPD*, steroid use, bleeding disorder, WBCs*, creatinine, albumin	Pre-operative	Defined depending on the complication. Examples are provided in the article.	Requires many variables. Severe complications were not defined according to the Clavien–Dindo definition. No pancreas specific variable.

ASA* American Society of Anesthesiology, BMI* Body Mass Index, DM* Diabetes Mellitus, WBCs* White Blood Cells, FRS* Fistula Risk Score, ACS-NSQIP* American College of Surgeons-National Surgical Quality Improvement Program, CHD* Coronary Heart Disease, COPD* Chronic Obstructive Pulmonary Disease. # Development study √ External validation study.

## Data Availability

Data can be provided anonymously when requested.
